# Supplementary material for: Optimizing clinical dosing of combination broadly neutralizing antibodies for HIV prevention
Source: PLoS Comput Biol. 2022 Apr 6;18(4):e1010003. doi: 10.1371/journal.pcbi.1010003 (PMC9084525; doi:10.1371/journal.pcbi.1010003)
Supplement: S1 Text — (PDF) [file pcbi.1010003.s001.pdf]

S1 Text: Optimizing clinical dosing of combination broadly neutralizing antibodies for HIV prevention

BT Mayer, AC deCamp, Y Huang, JT Schiffer, R Gottardo, PB Gilbert, DB Reeves

Additional notes on Hill function and titer

Many experiments involving broadly neutralizing antibodies (bNAbs) begin with a sample of an individuals serum after they have had a bNAb infusion. Thus the concentration of the antibody C_i is known. By modulating bNAb concentrations, the concentration at which an antibody neutralizes X% of a certain virus, or its ICX, can be defined directly. Note, neutralization is experimentally measured by fluorescence and typically IC50 and IC80 are quoted.

In this case, experimental neutralizations are fit to a Hill function, which we call the neutralization, which importantly specifies an interaction between a chosen bNAb i as well as a chosen HIV virus j

$$\nu_{ij} = \frac{1}{1 + \left(\frac{C_i}{IC50_{ij}}\right)^{-h}} \tag{S1}$$

Therefore, if $C_i \gg \mathrm{IC50}_{ij}$, this function goes to 1, if $C_i \ll \mathrm{IC50}_{ij}$, it goes to zero, and if $C_i = \mathrm{IC50}_{ij}$ it becomes 1/2. We refer to the ratio $\tau_{ij} = \frac{C_i}{IC50_{ij}}$ as titer. However, this quantity has also been called the inhibitory quotient.

However, for more generalized experiments attempting to quantify an antibody response (for example after vaccination), the concentration of the antibody is not known directly. Therefore, its potency can be quantified by beginning with a sample of plasma and then diluting, determining the dilution factor at which the serum neutralizes a certain virus by 50% compared to a control.

Accordingly, in this case the 'titer' is controlled completely by a scaling factor IDT, the inhibitory fold-dilution to achieve T% neutralization. Thus if the titer is 1000, there is a 1000-fold reduction of concentration relative to serum level, and neutralization obeys accordingly to our model Eq. S1, where we substitute in the unknown concentration of antibody of interest divided by this dilution factor, i.e. C_i/IDT. [1]

A useful property of this Hill model is that any two of three parameters can be used to infer the remaining. For example, Hill slope can be calculated as

$$h = \frac{\log 4}{\log(IC80/IC50)}. \tag{S2}$$

Therefore, if the Hill-slope is 1, a typical approximation and one that holds well for VRC01, then the ID50 and ID80 are related by a constant factor of 4.

[1] Note, sometimes the 'reciprocal dilution' $\delta_T = 1/\mathrm{IDT}$ is used, in which case quoting reciprocal dilution of 1/1000 would mean a 1000-fold reduction.

Or, if inhibitory concentration at a different threshold X is desired, this can be calculated as

$$\mathrm{ICX} = \mathrm{IC50}\left(\frac{X}{1-X}\right)^{1/h} \tag{S3}$$

By linearity, these formulae also apply to titration factors IDX, i.e.

$$\mathrm{ID80}/\mathrm{ID50} = 4^{-1/h} \tag{S4}$$

but note the minus sign in exponent related to reciprocal definition.

Instantaneous inhibitory concentration (IIP) is another valuable metric to distinguish the extremes of antibody potency. Because it is on the log-scale, it shows the difference between 99% and 99.9% potency, which can make a big difference when presented with a large/viral diverse population.

The general IIP is defined

$$IIP_{ij} = -\log_{10}[1-\nu_{ij}] \tag{S5}$$

which can also be neatly expressed in terms of titer as $\log_{10}[1+\tau_{ij}^h]$. However, as we will see calculating a combination titer requires additional steps for combination models.

Since neutralization is usually parameterized using IC50, one could convert from IC80 using Hill slope h as in Eq. S2.

Derivation of theoretical combination titration factors

We defined a *combination titer* (IDT) as the dilution factor applied to a sample of serum containing a bNAb combination that reduces neutralization to T%. Importantly, this implies all antibody concentrations are diluted by the same factor.

Additive titer

The 'additive' model assumes that the combined titer of multiple antibodies against a virus j is equal to their sum, i.e.

$$\tau_j^{add} = \sum_i \tau_{ij} \tag{S6}$$

Therefore, the combined neutralization under the additive model is

$$\nu_{ij}^{add} = \frac{1}{1+\left(\tau_j^{add}\right)^{-h}} \tag{S7}$$

such that we can call this neutralization after some dilution factor IDT

$$V_T = \frac{1}{1 + \left(\tau_j^{add}/\mathrm{IDT}\right)^h} \tag{S8}$$

We can then derive the titration factor associated with a combined neutralization of a certain level by inverting this equation:

$$\mathrm{IDT} = \left(\frac{V_T}{1 - V_T}\right)^{-1} \sum_i \tau_{ij}. \tag{S9}$$

And therefore, the factor scaling titer to drop neutralization by 50% naturally equates to the combined additive titer

$$ID50^{add} = \sum_i \tau_i. \tag{S10}$$

General Bliss-Hill (BH) titer

The 'Bliss-Hill' (BH) model assumes that each antibody in a combination works independently to neutralize a fraction of viral particles. This results in the formulation

$$\nu_j^{BH} = 1 - \prod_i (1 - \nu_{ij}) \tag{S11}$$

As above, we can solve for the scaling factor needed to drop the neutralization by a given factor by solving

$$V_T^{BH} = 1 - \prod_i \left[1 - \frac{1}{1 + \tau_{ij}/\mathrm{IDT}}\right] \tag{S12}$$

In general for combinations of more than 2 antibodies closed form solutions for IDT are difficult and numerical solutions are required.

Bliss-Hill titer with two bNAbs and $h = 1$

We now show the Bliss-Hill titration factor for two products with titers against the same virus (so dropping the j index, τ_1 and τ_2). We assume a Hill slope $h = 1$ to achieve a closed form solution. Beginning with Eq. S12, we expand out to

$$\frac{V_T}{1 - V_T} = \frac{\tau_1 \tau_2}{\mathrm{IDT}^2} + \frac{\tau_1 + \tau_2}{\mathrm{IDT}}. \tag{S13}$$

Which has a quadratic relationship. The solution follows:

$$\text{IDT} = \frac{2\tau_1\tau_2}{-(\tau_1+\tau_2) \pm \mathcal{D}} \tag{S14}$$

with the discriminant:

$$\mathcal{D} = \sqrt{(\tau_1+\tau_2)^2 + 4\tau_1\tau_2 V_T/(1-V_T)}. \tag{S15}$$

The solution using the negative of the discriminant is outside of biological observation and we consider only the positive solution.

Finally, we have

$$\text{ID50}^{BH} = \frac{2\tau_1\tau_2}{-(\tau_1+\tau_2) + \sqrt{(\tau_1+\tau_2)^2 + 4\tau_1\tau_2}} \tag{S16}$$

The important conclusion here is that depending on the given two titers at some time, the factor required to drop the combination neutralization by 50% is not trivial as in the additive model.

Differences in IIP: combination neutralization vs combination titer under BH model

Key concept: BH combination titer does not uniquely predict neutralization and/or IIP

For three of the models used in the manuscript, the minimum (worst case) the maximum (best single antibody) or the additive model (see section above), a combination titer is easily calculated from multiple individual antibody titers. It is the minimum, the maximum, or the sum respectively. However, this relationship is not trivial for the Bliss Hill model, which requires a nonlinear map from original titers into neutralization, makes the combination using the individual neutralizations (Eq. S11), and then in some instances this can be mapped back to a combination titer analytically (others numerically). That is, even assuming $h = 1$, adding individual IIP does not suffice to describe the combination titer (and therefore the combination IIP).

$$\sum_i \log_{10}[1+\tau_i] \neq \log_{10}[1+\tau_{ij}^{BH}] \tag{S17}$$

For example from the lhs, by specifying τ_1 and IIP=2, we can derive $\tau_2 = \frac{10^2}{(\tau_1+1)} - 1$. However, it can be seen immediately that substituting in this relationship results in a different value of Eq. S16, depending on τ_1. As a numerical example, $\tau_1 = \tau_2 = 9$ satisfies this relationship and leads to IIP=2. This implies two bNAbs 9-fold above their 50% threshold. It illustrates how potency gains are made by antibody combinations. However calculating the combined titer and substituting this into the rhs of Eq. S17 above leads to a substantial underestimate of potency: IIP = 1.36.

There is a mechanistic interpretation when calculating IIP using the combination neutralization derivation (Eq S27): the Bliss-Hill interaction is applied at the event-level first where a single combined neutralization estimate is generated and then translated through the IIP definition. On the other hand, the interpretation using the combination titer is different and effectively a distance metric: the combination titer is a factor applied to all concentrations that scales the current combined by 50%. The interpretation of this rescaling on the combination IIP-scale is not clear.

Again, this complexity only arises in the BH model (of the 4 we explored), the combined IIP can be equivalently calculated using either combination neutralization or the combination titer for the additivity, maximum, and minimum interactions.

This result has additional implications when considering correlates and protection. If a single bNAb trial suggests a titer correlate. For example, in the AMP studies, a titer of 100 (meaning VRC01 > 100-fold the exposed strain IC50) predicts prevention. If a BH model is to be used, this result must be translated carefully to predict similar protective titers for coming combination studies. As done in the empirical case study in the main manuscript, we derive independent protection estimates for each bNAb first, then apply the BH model to get a combined protection estimate. This is in contrast to an alternative strategy of calculating a combination titer first then calculating protection which could underestimate potency/protection.
