# Supplementary figures and images for: Optimizing clinical dosing of combination broadly neutralizing antibodies for HIV prevention
Source: PLoS Comput Biol. 2022 Apr 6;18(4):e1010003. doi: 10.1371/journal.pcbi.1010003 (PMC9084525; doi:10.1371/journal.pcbi.1010003)

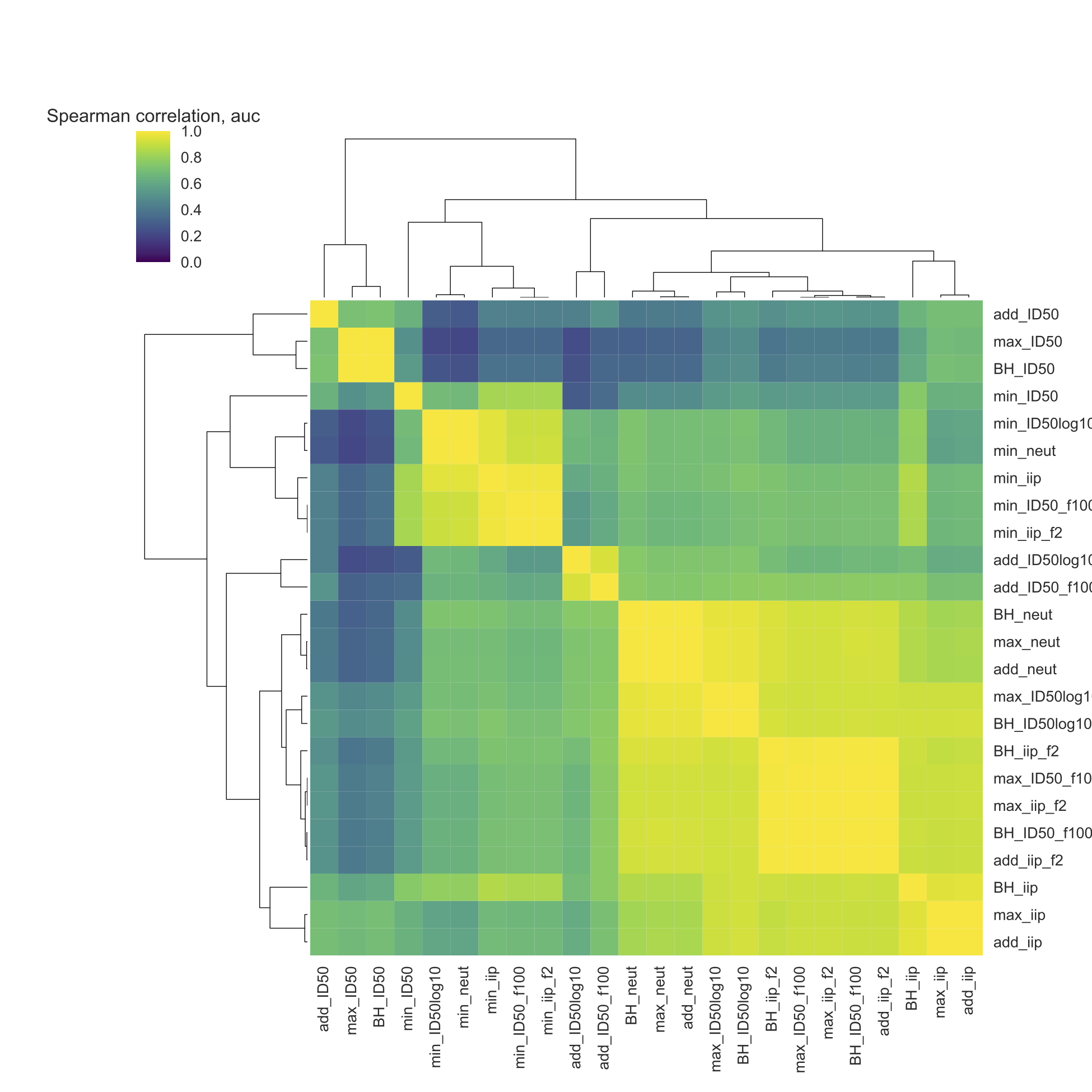

Supplement: S2 Fig — As for trough in Fig 2A, endpoints cluster by Spearman correlation into similar 5 main categories, from top to bottom: titer, minimum, additive titer, neutralization/coverage, and IIP. (TIF) [file pcbi.1010003.s003.tif]
