# Supplementary material for: Optimizing clinical dosing of combination broadly neutralizing antibodies for HIV prevention
Source: PLoS Comput Biol. 2022 Apr 6;18(4):e1010003. doi: 10.1371/journal.pcbi.1010003 (PMC9084525; doi:10.1371/journal.pcbi.1010003)
Supplement: S1 Table — (DOCX) [file pcbi.1010003.s007.docx]

**Table S1:** **Population PK input parameters for the empirical case study optimization**.

The -T variants derived to extend half-life based on PK analysis of parental variants.

| **parameter** | **10-1074-T** | **3BNC117-T** | **VRC07-523LS** |
| --- | --- | --- | --- |
| Vc | 4.50 | 5.02 | 1.89 |
| Cl^a^ | 0.09 | 0.22 | 0.09 |
| Q | 0.67 | 2.19 | 0.41 |
| Vp | 3.29 | 8.65 | 2.34 |
| ka^b^ | 0.39 | 0.39 | 0.39 |
| bioavailability^b^ | 0.46 | 0.46 | 0.46 |
| HL (days) | 64.57 | 45.62 | 35.76 |

^a^Clearance parameter derived for -T variants by dividing study-estimated clearance by three-fold to extend the half-life.

^b^Parameter data only available from [PMID: 31473167].
