# Supplementary material for: Optimizing clinical dosing of combination broadly neutralizing antibodies for HIV prevention
Source: PLoS Comput Biol. 2022 Apr 6;18(4):e1010003. doi: 10.1371/journal.pcbi.1010003 (PMC9084525; doi:10.1371/journal.pcbi.1010003)
Supplement: S2 Table — (DOCX) [file pcbi.1010003.s008.docx]

**Table S2: Ratio optimization results from 3-bNAb optimization for 3BNC117-T, 10-1074-T, and VRC07-523-LS.**

|  |  |  |  | **Optimal dosing proportion** | | |  |
| --- | --- | --- | --- | --- | --- | --- | --- |
| **% protection target** | **Time  endpoint** | **Study  correlate** | **% viral  coverage** | **3BNC117-T** | **10-1074-T** | **VRC07-523-LS** |  |
| 50% | AUC | AMP | 58 | 0 | 0.2 | 0.8 |  |
|  |  | NHP | 88 | 0.04 | 0.19 | 0.77 |  |
|  | trough | AMP | 40 | 0.03 | 0.24 | 0.73 |  |
|  |  | NHP | 78 | 0.07 | 0.2 | 0.72 |  |
| 95% | AUC | AMP | 24 | 0 | 0.46 | 0.54 |  |
|  |  | NHP | 49 | 0.01 | 0.27 | 0.72 |  |
|  | trough | AMP | 15 | 0.09 | 0.71 | 0.2 |  |
|  |  | NHP | 31 | 0.08 | 0.33 | 0.59 |  |
|  |  |  |  |  |  |  |  |
