# Supplementary material for: Optimizing clinical dosing of combination broadly neutralizing antibodies for HIV prevention
Source: PLoS Comput Biol. 2022 Apr 6;18(4):e1010003. doi: 10.1371/journal.pcbi.1010003 (PMC9084525; doi:10.1371/journal.pcbi.1010003)
Supplement: Attachment — Submitted filename: bnabcombo-reviewresponse.docx [file pcbi.1010003.s009.docx]

**We are very grateful for the efforts of the reviewers and have made changes based on the constructive critiques. We respond to all comments below in a point-by-point fashion in bold.**

Reviewer #1:

Mayer et al provide an important framework for considering what ratio of bNAbs within a multi-bNAb cocktail is optimal, given a single dose of fixed size. The model builds upon prior work in the field and incorporates PK, PD and interactions between bNAbs under multiple interaction scenarios. Useful case-studies comparing dose considerations for a bispecific vs 2-bNAb combination, and consideration of various bNAb ratios within a clinically relevant 3-bNAb cocktail, are illustrative. The analyses appear very well developed and comprehensive in scope. The author’s insights, in addition to their publicly available web-based tool for ratio optimization, should benefit investigators when planning future clinical or preclinical studies to evaluate prophylactic or therapeutic combinations of bNAbs.

Addressing the following minor comments should help improve the clarity and readability of the manuscript:
Line 44: The sentence beginning “Less sensitive variants . . .” is unclear and appears to state the opposite of what was stated two sentences earlier.
Line 46: typo (PrEP)
Line52: Please check accuracy of reference 12. Also, the Schiffer, et al reference is listed twice in the bibliography (as ref 12 and 26)
Lines 449-450: Please rephrase the sentence beginning “To calculation IIP under Bliss-Hill . . .” for clarity.

**We thank the reviewer for their effort reviewing our paper and their supportive comments. We have made all their suggested edits.**

Reviewer #2:

1. This framework adds exceptionally to the field and enables design of future combination antibody studies. Furthermore, this tool can be used to enable more robust phase 2/3 study designs.

**Thanks! We really appreciate this and hope the tool will be used for these important decisions going forward.**

2. PK modelling for the AMP trial was pivotal for dose selection which predicted the target bNAb concentrations. Unfortunately, the assumptions about sensitivity/early resistance in the population were off target. It would be great to see examples of how the authors use the clinical data as an input into the system to enable the readers to see the comparison of how the pre-clinical data versus the actual clinical data translates

**This is an excellent suggestion. We’ve now added protection estimates derived from the AMP trials to our 3-bNAb combination case study for direct comparison with the results from the NHP SHIV challenge study meta-analysis. The AMP results are less optimistic, and we highlight that there is some sensitivity to the optimal design based on the underlying protection estimates.**

3. The authors highlight practical considerations about dosing. It is important to address the other complexities and challenges with the use of combination products. A single co-formulated drug product is ideal but will also need to take into account the specific formulations etc. It would be nice to have additional characteristics such as viscosity and concentration formulations inputted into the system to determine if the products can be co-formulated as well.

**We agree that these manufacturing considerations are of critical importance for the viability of the product regimen. From the PK perspective, these types of issues would be assessed in Phase 1 study of co-formulated administration and should be prior information determining half-life and distribution. It is specifically interesting in the PD framework because these considerations on *in vitro* protection are unknown, however the uncertainty is hard to quantify generally besides a specifically tailored simulation sensitivity analysis. We borrow some of this language and introduce these ideas into the the Discussion paragraph of the bi-specific case-study, explaining that there are potential trade-offs using co-formulated products.**

4. The authors mention limitations “although most of our analysis concerns prevention studies, this framework is applicable to curative studies attempting to use bnAbs to prevent viral rebound after stopping ART-within-host diversity in the reservoir is the challenge here.” Previously described literature suggests use of predictive modelling based on env sequencing of the individual’s quasispecies to determine bnAb sensitivity patterns and optimize combination cocktails, including scoring AA signature types for bnAb resistance, prior to using a particular bnAb. Refer to Magaret CA, Benkeser DC, Williamson BD, Borate BR, Carpp LN, Georgiev IS, et al. Prediction of VRC01 Neutralization Sensitivity by HIV-1 Gp160 Sequence Features. PloS Comput Biol (2019) 15:e1006952. doi: 10.1371/journal.pcbi.1006952. Have the authors considered adding these features to the existing model?

**This is a very interesting question for the extension of this framework. The modeling framework presented in the manuscript predicting potential bNAb regimen prevention efficacy includes as inputs IC50s of each bNAb clinical product in a regimen against a panel of HIV-1 Envelopes that are representative of the expected distribution of circulating strains in a future prevention efficacy trial. Therefore, the larger the sample size of IC50s, the greater precision of the modeling outputs. Magaret et al. (2019) and Bricault et al. (2019, Cell Host & Microbe) showed that there is information in Env sequences for partially predicting IC50 against bNAbs, and that predicted IC50s can be calculated, with quantification of prediction accuracy, using the SLAPNAP tool that is publicly available (Williamson et al., 2021, *Bioinformatics*). If circulating viruses for all or part of the study population planned for a future prevention efficacy trial has limited direct data available on IC50s yet there is a much larger set of Env sequences available representing those circulating viruses (a common situation given the greater laboriousness in neutralization experiments), then the IC50 inputs may be augmented with predicted IC50 inputs to increase the precision of the modeling. We now mention this concept in the discussion.**

**On the question of application of the modeling for curative studies, a panel of within-host quasispecies could serve as the neutralization panel to be used in assessing combinations for effectiveness within host. However, as the reviewer cites and in a recent preprint (**https://www.medrxiv.org/content/10.1101/2021.12.03.21267269v1.full.pdf**) suggests, a more complicated neutralization framework might be necessary to model viremia suppression and rebound. Very little work has been done on modeling bNAbs and HIV cure, and we expect the undersampling issues associated with HIV reservoirs will be a significant barrier to effectively assessing diversity and the chance of escape/rebound. We believe the basic framework we outline here (as depicted in Fig. 1) can serve as a basis for a cure design, but there are a great number of nuances that deserve their own elaboration in future work on cure.**

Reviewer #3:

The manuscript uses theoretical model to evaluate the effect of combination of monoclonal antibodies on the outcome of anti-HIV therapy. The work being evaluated is very relevant and mathematical modeling and simulation can provide great insight into the goal. However, unlike authors’ previous work (i.e., ref # 18 and 19) the work presented here is purely speculative, and add little to the field. Either most conclusions are obvious, or the analysis is inclusive.

**We really appreciate the reviewer reading this and our prior work, though we respectfully disagree with some of these statements, and we respond in the following:**

On work presented here is purely speculative and adds little to the field:

**-We believe our framing research question: “At a fixed dose, how do we define the best ratio of multiple antibodies?” is a critical consideration when designing a clinical trial with multiple products. Of note, this specific question is not addressed in any prior work (including cornerstone work by Wagh, Korber et al.)**

**-In particular, the 3-bNAb design (fig 4) represents a practical example of how to apply our tool to a clinical design that will likely be tested soon
-As Reviewers 1 and 2 point out, the web-based tool provides investigators a hands-on interface such that optimization before a clinical trial can be explored. This tool allows investigators who may have more direct insight to input their own definitions of what it means for bNAbs to perform well in a certain context (e.g., do you seek breadth against strains throughout, or really high potency at trough? etc.)
-PKPD modeling is a rather diffuse field, and we believe our exposition here could be a important resource for viral dynamic modelers who hope to add models of drugs.**

**- Moreover, we have performed novel calculations (to the best of our knowledge) such as the mathematical derivation of combination neutralization titer endpoints when concentrations are not fixed as described in Table 1. These calculations are actively being applied to the combination bNAb regimen studied in HVTN130/HPTN089 to compare predicted vs. experiment combination titer measurements. While these data are unpublished, these results will be presented at CROI this year.***

*Magdalena E. Sobieszczyk, Sharon Mannheimer, Yunda Huang, Carmen A. Paez, Theresa Gamble, Deborah A. Theodore, Wairimu Chege, Margaret Yacovone^5^, Chenchen Yu, Lily Zhang^3^, Jack Heptinstall^6^, Kelly Seaton, Georgia Tomaras, David C. Montefiori, the HVTN 130/HPTN 089 Study Team. PHASE 1 STUDY OF COMBINATION ANTI-HIV NEUTRALIZING ANTIBODIES IN HIV NEGATIVE ADULTS. CROI 2022. *Accepted*.

On conclusions being obvious:

**We are unsure which conclusions were obvious to the reviewer. We were not surprised that many outcomes clustered together, but it was quite surprising to us that optimization is highly dependent on outcome choice and that PK has such a high degree of influence on overall bNAb neutralization/optimization choices, especially given that it has not been previously integrated into models.**

On analysis inclusive:

**While it was a philosophical choice to include many possibilities, the design of the simulations was guided by conversations with investigators who conducted the AMP trials and are planning future bNAb HIV prevention efficacy trials.**

In addition, the author use 1-compartment PK model with 3L volume to capture antibody PK, which is bound to provide higher than actual contractions.

**Thank you for this useful question. Directly compared to a 2-compartment model, the 1-compartment model will have higher AUC and trough owing to a lack of distribution phase. However, we note the sensitivity analysis assesses the correlation of the results, not the absolute point estimates so the over-estimation is not a direct concern here. Throughout the manuscript, when we use a 1-compartment model, the results are interpreted relatively. However, based on this comment, we did remove the 2400 mg/kg dose from the sensitivity analysis so as to not have an overly optimistic set of results. The results were not notably affected.**

**In the context of the bi-specific, we only note that PK is an important component of the system and do not explicitly comment on the absolute performance of the theoretical candidate. For the 3-bNAb combination, the closest to a realistic trial optimization and the only situation in which we report protection estimates, we use a complete 2-compartment absorption model specified from Phase I studies.**

**In summary, we believe we have properly leveraged the simplified PK model in the sections to gain intuition for our model framework. For the part of the paper where precision matters, we use the fully specified PK model.**

**One final technical note: for the volume specifically, the dose and distributional (or central) volume are non-identifiable with respect to PK. Specifically the AUC and trough can be scaled linearly if the other PK parameters are fixed. So if the volume were truly higher (e.g., 2-fold higher or 6L) this could be assessed equivalently as a decrease in the input dose resulting in identical PK. Based on the Davda et al. (MAbs. 2014) meta-analysis and ranges from other Phase I HIV bnab studies, we believe we are within 2-fold of volume range and our dose range spans 10-fold.**

Also, the assumption that bi-specific antibody would have shorter half-life is not well-founded.

**We agree that our framing of the bi-specific was presented too strongly. To address this, we added additional references in the Discussion and Results motivating the example and highlighted more specifically the importance of PK in the context of neutralization for bi-specific antibodies. As the reviewer suggests, poor PK with a synthetic bi-specific antibody would be a speculation; and co-administration of multiple products has its own manufacturing limitations as suggested by Reviewer 2. The PK consideration and suggested caution is most relevant to the general message of the paper.**

Rather than simulative 4 different interaction conditions, may be in vitro work can be used to choose most realistic nature of combination.

**We agree that an *in vivo* study of protection would be critical to determine this but that such a study would likely to be a Phase II study and would need to be designed before this information is known. This is effectively the motivation for exploring both the additivity and BH interactions in the sensitivity analysis. Importantly, we show that Bliss-Hill and additive interactions (currently suggested by *in vitro* models [Wagh et al. 2016 and 2018] noted in the manuscript as well) tend to result in similar optimizations, but the “minimum” pushed to different conclusions.**

Lastly, there needs to be some comparison of model simulation with realistic data (e.g., clinical PK of mAb, viral dynamic data etc.) to provide confidence in the model and its output.

**To address this comment (and similar comment from Reviewer #2), we incorporated the AMP results directly in addition to the NHP data presented in the original submission. Unlike most other bNAb modeling studies, we now apply a clinical correlate to link in vitro potency estimates to in vivo protection. We believe this has strengthened the 3-bNAb regime case-study − which already incorporated realistic input settings −and are able to compare conclusion sensitivity based on these inputs.**

**The AMP studies were a first-in-kind proof of concept, and while we now have a critical “data point” to address protection uncertainty, there remain no studies of combination antibodies for HIV prevention. As we mentioned earlier, our titer calculations are also being validated through comparisons of experimental titer and predicted titer in the HVTN130/HPTN 089 combination antibody study. The link between viral load and prevention endpoints, as simulated in our prior work, has not been investigated yet and we are actively working on this which will be the focus of a future paper.**

Lastly, the manuscript is written in a way that the reader can get lost very easily if not familiar with authors’ previous work. It may be worth rewriting some part of the paper.

**We have made minor edits throughout for clarity and succinctness. Additionally, since the additive and maximum interaction models produced very similar results, we have removed the maximum interaction from figures/results to simplify. In addition to specifically responding to comments above, we believe this has strengthened the clarity and message of the manuscript and addressed this final, general comment.**
